# A Portrait of the Chromophore as a Young System—Quantum-Derived Force Field Unraveling Solvent Reorganization upon Optical Excitation of Cyclocurcumin Derivatives

**DOI:** 10.3390/molecules29081752

**Published:** 2024-04-12

**Authors:** Raúl Losantos, Giacomo Prampolini, Antonio Monari

**Affiliations:** 1 Departamento de Química, Instituto de Investigación en Química (IQUR), Universidad de La Rioja, Madre de Dios 53, 26006 Logroño, Spain; 2 ITODYS, Université Paris Cité and CNRS, F-75006 Paris, France; 3 Istituto di Chimica dei Composti Organo Metallici (ICCOM-CNR), Area della Ricerca, Via G. Moruzzi 1, I-56124 Pisa, Italy; giacomo.prampolini@pi.iccom.cnr.it

**Keywords:** cyclocurcumin derivatives, molecular photoswitches, solvent reorganization, quantum derived force field, molecular dynamics

## Abstract

The study of fast non-equilibrium solvent relaxation in organic chromophores is still challenging for molecular modeling and simulation approaches, and is often overlooked, even in the case of non-adiabatic dynamics simulations. Yet, especially in the case of photoswitches, the interaction with the environment can strongly modulate the photophysical outcomes. To unravel such a delicate interplay, in the present contribution we resorted to a mixed quantum–classical approach, based on quantum mechanically derived force fields. The main task is to rationalize the solvent reorganization pathways in chromophores derived from cyclocurcumin, which are suitable for light-activated chemotherapy to destabilize cellular lipid membranes. The accurate and reliable decryption delivered by the quantum-derived force fields points to important differences in the solvent’s reorganization, in terms of both structure and time scale evolution.

## 1. Introduction

In recent years, the interest towards molecular photoswitches, i.e., compounds which can undergo important structural and conformational reorganization upon (visible) light excitation has increased considerably. Indeed, in addition to their rich inherent photochemistry, molecular photoswitches may find suitable application in high added-value fields, including luminescent devices, smart materials, chemical or diagnostic probes, and even photodynamic and light-activated therapy [1,2,3,4]. Furthermore, natural occurring photoswitches, such as retinal photoswitches embedded in rhodopsin, are responsible for crucial biological functions, notably those related to signal transduction and vision [5,6,7].

As far as their use for therapeutic purposes is concerned, molecular photoswitches are particularly attractive, because they can overcome one of the main limitations of conventional photodynamic therapy (PDT) [8,9]. Indeed, PDT is based on the administration, either topically or systemically, of a drug which is inert and non-toxic in the dark but undergoes energy transfer with molecular oxygen triggered by the absorption of a suitable wavelength, to produce the highly toxic singlet oxygen. This photochemical pathway leads to the destruction of biological macrosystems, and thus cell death. As a matter of fact, PDT has found suitable application in cancer therapy [10,11,12,13,14], as well as in the fight against bacterial or viral infection [15,16], or inflammatory diseases such as psoriasis [17,18]. The advantage of PDT is due to the spatial and temporal selectivity brought by the light activation, which significantly diminishes adverse systemic side-effects, especially compared to conventional chemotherapy [12,19,20]. Yet, the necessity to rely on singlet oxygen limits its application to solid tumors, which usually contain hypoxic conditions [21].

The most attractive alternative to maintain the light activation selectivity would be to rely on molecular photoswitches, which, by undergoing important conformational changes due to light excitation, would destabilize biological macrostructures, such as proteins or lipid membranes, and hence trigger cellular apoptosis or necrosis. These techniques, which can be grouped under the general name of light-activated chemotherapy (LAC), are becoming increasingly popular, and different proposals for suitable agents have been reported [9,22,23]. Recently, the use of unidirectional molecular motors [24] or molecular plasmons [25] have been suggested. Photoswitches interacting with neural receptors or serotonin transporters have been proposed and may also affect the glooming field of optogenetic regulations [26,27]. Nonetheless, for its effective use in a LAC framework, a molecular photoswitch should possess some important properties, namely absorption in the visible and infrared portion of the electromagnetic spectrum (ideally in the therapeutic window), a controlled, environment-independent photoisomerization, always characterized by a high quantum yield, and finally, the capacity to lead to important structural perturbations of the biological system because of its isomerization. Its non-toxicity in the dark, as well as its solubility and bioavailability, are also crucial to assure the potential use of the chromophore as a LAC agent. The use of natural or bioinspired compounds as chromophores for LAC or PDT purposes is a promising strategy, which could also help minimize the toxicity of the proposed drugs in the dark. Extracts from Turmerica longa (turmeric), such as curcumin, have found suitable applications in PDT, due to their capacity to generate singlet oxygen, combined with the inherent antimicrobial and anti-inflammatory properties of curcumin [28,29,30,31]. As a matter of fact, and thanks to its use as a spice, curcumin has been proposed for the light-induced sanitization and treatment of food and edible meat [32].

**Figure 1:**
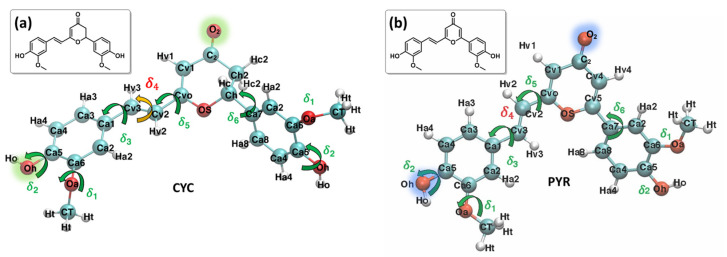
Most stable conformers and QMD-FF labels employed for each system: CYC, panel (**a**), and PYR, panel (**b**). Selected atoms, relevant to the following discussion, are highlighted in green and blue.

The photophysics of curcumin and the interplay between intersystem crossing and other relaxation mechanisms, including hydrogen transfer, have also been reported recently using non-adiabatic surface hopping dynamics [31]. However, it has been shown that a secondary component of turmeric, cyclocurcumin (CYC, Figure 1), can undergo reversible trans/cis photoisomerization after the absorption of visible light [33]. Therefore, it could be regarded as a promising agent for LAC, also because of its non-negligible two-photon absorption cross-section, which could shift its activation to the therapeutic window [34]. However, the photoisomerization quantum yield of natural CYC is strongly dependent on the embedding environment; this channel is quenched in polar solvents [33,34]. Therefore, we have recently proposed, synthesized, and characterized a γ-pyrone cyclocurcumin (PYR, Figure 1) analogue, which has red-shifted absorption and favorable photoisomerization characteristics, regardless of the polarity of the medium [35,36]. Its interaction with biological lipid membranes has also been studied, both experimentally and theoretically; its capacity to perturb the membrane has been identified, and, thus, it can potentially induce apoptosis [37,38,39].

Methodologically, the study of photoisomerization in complex environments should require the use of proper multiscale approaches, tackling, for a sufficient time interval, the subtle interplay between different phenomena, such as electronic structure and conformational reorganizations. As shown by the previous examples and by the behavior of natural CYC, the interaction with the solvent, its reorganization following the electronic excitation, and eventually the non-adiabatic relaxation, are fundamental to rationalize the outcome of the light-induced processes [34]. This is particularly true when the chromophore is meant to interact with strongly inhomogeneous biological environments, such as lipid membranes characterized by a hydrophobic core flanked by polar heads. Yet, identifying the precise coupling between the reorganization of the environment and the probe’s electronic excitation opens critical methodological challenges, which should be properly considered. The highly successful 50-year-old continuum approaches, such as polarizable continuum methods [40,41], which are rooted in equilibrating an average continuum distribution to the chromophore electronic density, fail to capture the non-equilibrium excitation and the anisotropic reorganization.

Non-adiabatic dynamic methods, either in the semi-classical surface hopping approach or based on the propagation of nuclear wavepackets, have achieved impressive results in rationalizing and describing complex photochemical and photophysical processes [42,43], also including spin–orbit coupling and arbitrary external perturbations [44,45]. As a most paradigmatic example, we may cite the pioneering studies on the isomerization of retinal photoswitches embedded in rhodopsin, which is at the heart of the visual process [46,47]. The development of a non-adiabatic dynamics method to assess the time evolution of the excited state manifolds in complex environments is actively being pursued, including the optimization of QM/MM approaches [48,49], the inclusion of polarizable force fields [50], the use of semi-empirical approaches [51], or machine learning approaches [52]. Furthermore, interesting approaches, such as the linear vibronic coupling surface-hopping dynamics approach [53,54], have emerged for satisfactorily describing the photophysics of rigid metal complexes [55,56], yet they are not adapted in the presence of highly flexible and anharmonic degrees of freedom, such as those activated in molecular photoswitches, based on flexible organic probes.

A complementary approach, which will be followed in the present contribution, relies on the use of classical molecular dynamics (MD), in which the lack of standard force-fields (FFs) able to describe the specific electronic states of the chromophore is overcome by resorting to quantum mechanically derived force fields (QMD-FFs), here obtained by the Joyce procedure [57,58]. In this approach, the general transferability of conventional universal FFs is abandoned in favor of an accurate and specifically tailored description of the target chromophore [59,60,61], for which the geometrical parameters and the atomic point charges that are specific to the different excited states can be derived from a reliable QM description. It is worth noticing that the QMD-FF expressions can range from the simple forms employed in general purpose FFs to more complex models, yet they always retain the computational convenience of standard MDs. The resulting ground and excited state QMD-FFs are thereafter exploited in classical MD simulations, to explore the potential energy surface and the conformational space of a given adiabatic state. This allows for including an explicit description of the solvent, modelled by classical FFs, while simulating the light excitation by switching the target chromophore’s QMD-FF from the one specifically modeled for the ground state (S_0_) to that obtained for an excited electronic state (S_1_). Following this procedure, one can thus perform a non-equilibrium MD (neqMD), repeated on a statistically significant set of different initial conditions, which eventually allows for the description of the solvent early relaxation, which is potentially crucial to determine the ensuing photochemical pathways.

In this contribution, we parameterize the ground and first excited states of the natural CYC and the biomimetic PYR via the Joyce QMD-FF. After validating the accuracy of the resulting FF description, with respect to its capacity to reproduce the structural and optical properties of the chromophore as described by density functional theory (DFT) results, neqMD simulations confirm a different behavior of the water environment in the solvent relaxation pathways of the natural and the biomimetic chromophores.

## 2. Results and Discussion

The Joyce procedure was carried out for natural CYC and the biomimetic PYR by the usual two-step procedure [57,58], achieving a final standard deviation of 0.893, 1.033, and 0.288, 0.304 kJ/mol, for S_0_ and S_1_, respectively. Concretely, for each molecule/state, all harmonic stiff modes were first parameterized at once, based on the QM Hessian matrix. Thereafter, the parameters for a reliable description of the most flexible degrees of freedom (shown in Figure 1) were obtained using the Frozen Internal Rotation Approximation (FIRA), as implemented in Joyce [62,63]. Finally, to model the presence of coupled internal coordinates, we resorted to specific non-bonded intra-molecular LJ interactions, as in previous applications [64], which have been included for the coupling between the rotation of HO and OMe groups on both aromatic rings, i.e., the δ_1_ and δ_2_ coordinates, as shown in Figure 1.

**Figure 2:**
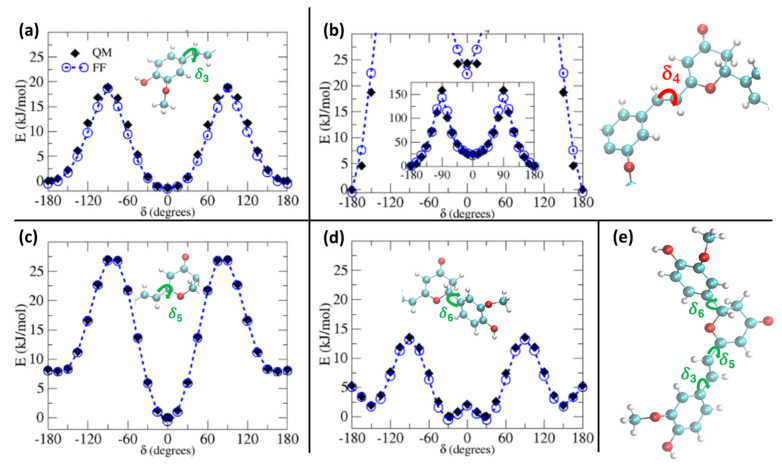
S_0_ relaxed torsional profiles computed for PYR at QM (black diamonds) and QMD-FF (blue circles) level. Scans considering the dihedrals δ_3_ to δ_6_ (see insets) are shown in panels (**a**–**d**), respectively, whereas in panel (**e**) the definition of the flexible dihedrals is again displayed for reference.

In Figure 2, we report the comparison between QM and QMD-FF torsional energy relaxed scans (see Methodology) for the soft degrees of freedom for the ground state of PYR. To simplify the main text and keep the focus on the procedure, without indulging into unnecessary details, we have moved to the Supplementary Materials the outcomes of the relaxed scans along the soft internal coordinates of the natural CYC, as the results are very similar to the ones hereafter shown for PYR. In addition, the presence of coupled internal coordinates, like for δ_1_ and δ_2_, would require a 2D scan to be correctly compared. Here, for the sake of clarity, we report a section of the 2D surface, in which one of the coordinates is kept constant at a given value, while the other one is varied, as can be appreciated in Supplementary Materials. As observed in Figure 2, the QMD-FF-generated PES almost completely overlaps with the QM values, confirming that the FF can properly account for the molecular displacement, also concerning the most relevant, flexible, and non-harmonic degrees of freedom.

**Figure 3:**
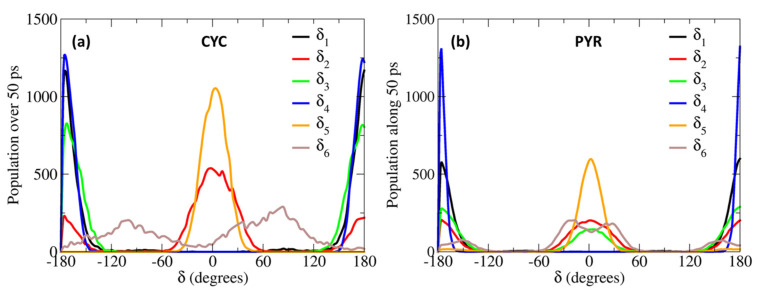
Population distributions of the most flexible dihedrals, computed along the 50 ps MD trajectories, obtained for CYC (**a**) and PYR (**b**), in water solvent.

The larger difference is observed for the rotation of the δ_4_ dihedral, for which the QMD-FF slightly underestimates the maxima. However, it should be noted that δ4 defines the rotation around the Cv2=Cv3 double bond and is not expected to show the same degree of flexibility as the other dihedrals. In fact, considering the significantly steeper profile and the unfavorable energies associated with its maxima (~150 kcal/mol), it is highly unlikely that these PES regions will be visited during the MD simulation at room temperature. To further validate our QMD-FF for the remaining stiff coordinates, we also compared the calculated harmonic vibrational frequencies with the results obtained at the DFT level; as shown in Supplementary Materials, only negligible deviations are observed for both systems. Thus, we may consider that our ground state QMD-FF successfully reproduces the molecular behavior in terms of vibrational modes, rotation energies, and geometrical conformations. After the proper equilibration of each solvent, obtained as described in the previous section, a series of NPT dynamics was carried out for CYC and PYR in water, acetonitrile, and ethanol, simulating all systems up to 50 ns, at ambient conditions. This also allows for further validating the stability of the parameterized QMD-FFs. To gain a deeper insight into the probe’s conformational dynamics, we have monitored the most relevant flexible coordinates considered during the parametrization procedure. The population distribution of the four dihedrals for the two chromophores in water can be appreciated in Figure 3. The other solvents yielded similar results, as presented in Supplementary Materials. The distribution of the dihedrals shows a rather similar behavior for both chromophores, the most remarkable difference being in the rotation along δ_6_ (see Figure 1 for definition). In agreement with the computed QM torsional profiles (see Figure 2 and Figure S2 in the Supplementary Materials), this soft internal coordinate appears to be more flexible for CYC with respect to PYR, and more frequent exchanges among the +/−60° and +/−150° minima are evident in the former chromophore.

This behavior can be traced back to the structural differences between CYC and its synthetic analogue, induced by the removal of one Hc2 proton, and the consequent strengthening of the involved C-C bonds, the increased π character of which allows for extending the conjugation to the neighboring rings (and partially to the Cv2=Cv3 double bond), hence enforcing the planarity of the aggregate. Interestingly, such electronic delocalization also causes additional minor differences, detectable in the δ_2_ and δ_3_ dihedrals. As far as the latter is concerned, the rotation of the second peripheral aromatic group (left side of the chromophore in Figure 1) is slightly favored in PYR, where the barrier of 19 kJ/mol separating the 0° and 180° degenerate minima is more easily overcome with respect to the one registered in CYC (23 kJ/mol). Turning to δ_2_, which drives the rotation of the C-OH bond, hence directly influencing the establishment of hydrogen bonds with the solvent, we found a more pronounced rotation of the OH moiety in CYC, leading to the population of both 0° and 180° minima, while in the case of PYR, the intramolecular hydrogen-bonded conformation around 0° is largely dominant.

**Figure 4:**
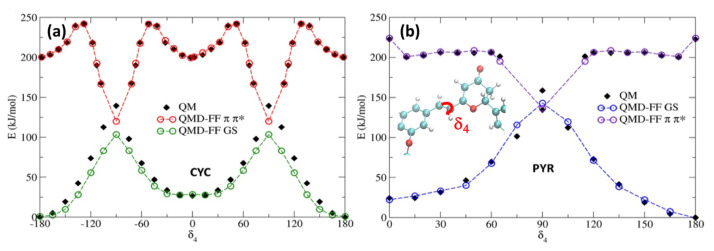
Potential energy profiles for the GS and π-π* states along the δ_4_. dihedral. Note that QMD-FFs for the π-π* state have been shifted by 200 kJ/mol to ease the comparison. (**a**) CYC; (**b**) PYR.

To account for the excited state solvent reorganization, we first generated a QMD-FF modeling the chromophore’s PES of the S_1_ bright π-π* singlet excited state. For this aim, we have repeated the same strategy used for S_0_, obtaining the π-π* minimum geometry, together with the corresponding Hessian, thus describing the vibrational normal modes at the TD-CAM-B3LYP/6-311++G** level. Conversely, we have decided to neglect the description of the n-π* state, since the latter is darker, and usually higher in energy than the π-π* state, especially for PYR. For both chromophores, the rotation around the isomerizable double bond (δ_4_) is challenging to be properly described by the TD-DFT approach used, since the potential energy of the π-π* state encounters a conical intersection with the ground state. As we had previously observed [34,36], the isomerization profile cannot be fully reproduced using a TD-DFT approach. The large coupling between both the ground and excited state in the region close to the conical intersection leads to a failure in the SCF, which is unable to converge in the main part of the geometries visited along the scan. A possible strategy to circumvent this issue is to specifically derive QMD-FF parameters for such problematic degrees of freedom by relying on a higher-level QM technique, while preserving the reference TD-DFT description for the Hessian matrix and the remaining soft coordinates for the sake of computational feasibility. Therefore, we have used our previously computed MS-CASPT2 δ_4_ profiles for each chromophore, again following the π-π* state. The potential energy surface scan along the δ_4_ rotation, obtained by sampling the profile at MS-CASPT2 level in steps of 15° [34], was first fitted with an “in house” code using the model’s FF model function for flexible coordinates (i.e., a Fourier series of cosine terms; see Equation (S9) in the Supplementary Materials). The resulting QMD-FF parameters were assigned during the subsequent Joyce parameterization, which was employed to find the best parameters for all other degrees of freedom, based on the QM data computed at the TD-DFT level. In Figure 4, we report the potential energy surface around the isomerizable double bond, for both chromophores, comparing the results obtained with the QMD-FF and at the QM level. As it has already been observed for the ground state, the agreement between the QMD-FF and QM energies is highly remarkable and clearly able to capture the behavior of this crucial degree of freedom, while maintaining an accurate description of the rest of the internal coordinates (see Supplementary Materials).

As previously reported [34,36], the isomerization of cyclocurcumin-based compounds is due to the population and evolution of the π-π* state. However, significant differences can be appreciated between the PESs of CYC and PYR. Indeed, CYC presents a high barrier of 42 kJ/mol separating the Franck–Condon region and the conical intersection, and hence the photoisomerization is slow and less efficient. Contrarily, in the case of PYR, the conical intersection can be reached, overcoming only a small barrier of 7 kJ/mol. Because of the higher barrier, it is thus unlikely that CYC may explore the dihedral close to 90 degrees; therefore, we have only extrapolated the same behavior to the 0 to 180 rotation, assuming a symmetrical potential energy surface. On the other hand, some assumptions have to be made in the case of PYR to correctly reproduce the shape of the PES by fitting the QM data. Specifically, we have hypothesized a symmetric behavior between the region [0°, 90°] and [90°, 180°], and thus covered the full periodic rotation. This was necessary, since the low barrier may lead to the population of regions close to the conical intersection (~90°); thus, points that are distant from the Franck–Condon geometry should be properly described.

Additionally, since we have not parameterized the [−180°, 0°] range, to avoid issues related to the dihedral sign degeneracy, we only allow rotations in the clockwise direction to occur, due to the use of a harmonic potential in combination with the polynomial cosine functions. However, this approximation should not alter the modeling of the physical properties of PYR, since the clockwise and anticlockwise rotations should be symmetric. As shown in Supplementary Materials, the scan for the other soft degrees of freedom performed in the excited state at the QMD-FF level are also perfectly overlapping with the results obtained with QM. The same is also true for the agreement between QM- and QMD-FF-based vibrational frequencies. Thus, the soundness of our approach is further justified.

**Figure 5:**
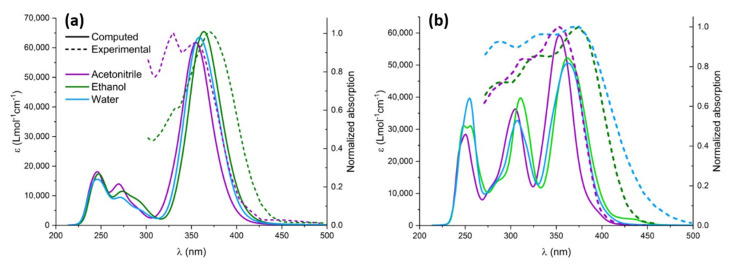
Experimental (dashed lines) and computed (solid lines) UV-Vis spectra in different solvents: acetonitrile (purple), ethanol (green), and water (blue). Computed spectra were redshifted by 14 nm to highlight solvatochromism. (**a**) CYC and (**b**) PYR.

After the proper validation of the excited state QMD-FF, we now turn, on the one hand, to the simulation of the absorption spectrum, focusing in particular on the effect of explicit solvation, and, on the other hand, to the geometrical evolution of the chromophore after the excitation and subsequent solvent relaxation, modeled through neqMD. To explicitly account for the solvent effects, we have extracted a series of non-correlated snapshots from the equilibrium ground state dynamics, containing the chromophore and a shell of neighboring solvent molecules. Specifically, we considered all solvent molecules laying inside a 4 Å-radius sphere with respect to the center of mass of the chromophore, in addition to the molecules found within 2.5 Å from the chromophore’s Oh, Oa, O_2_, and Ho atoms, which have been explicitly included in the TD-DFT calculations. The rest of the solvent was considered as a polarizable continuum modeled by the CPCM solvation procedure. The computed spectra for the different solvents are obtained by convoluting all the vertical transitions for all the 500 snapshots using a pseudo-Voigt representation with 75% Gaussian and 25% Lorentzian functions, normalized by the number of snapshots. As can be seen in Figure 5, both chromophores present a moderate solvatochromism and show an intense band, peaking at around 380 nm and extending to the blue region of the electromagnetic spectrum, which compares well with the experimental results. Unsurprisingly, the lowest energy absorption band is dominated by the transition to the bright π-π* state, while the influence of the n-π * transition is negligible due to the difference in their oscillator strength. Natural transition orbitals describing the electronic density reorganization of the two lowest singlet excited states are presented in Supplementary Materials.

The impact of solvatochromism is less important for CYC, although ethanol provides the most red-shifted absorption spectrum. Interestingly, and because of the different topology of the excited state potential energy surface, the photoisomerization of CYC is instead strongly solvent-dependent. In contrast, for PYR we may observe a notable blueshift in the case of acetonitrile, for which the absorption maximum is displaced at 350 nm. The peculiar behavior of the acetonitrile solution has also been observed experimentally and is nicely captured by our approach, which points out the subtle equilibrium between structural and electronic effects. Furthermore, and apart from acetonitrile, the solvatochromism as a function of the solvent polarity is well reproduced for the ensemble of the solvents, as compared to the experimental results [35]. Furthermore, in the case of PYR, we may observe a more complex spectra due to the larger contribution of the extended π cloud with respect to CYC, for which only a single peak may be observed.

To analyze the effects of the electronic density rearrangement following the electronic excitation, we mimicked the light absorption through a switch of the QMD-FF describing the chromophore ground state to the one specific to the π-π* state, and thereafter performed a non-equilibrium MD simulation. Our approach will allow for a complete study of the coupling between the solvent and the chromophore electronic reorganization, as represented by the differential QMD-FF point charges, and the nuclear degrees of freedom. To ensure statistical significance, a total of 500 non-correlated snapshots have been selected, together with their corresponding velocities, from the 50 ns ground state production trajectory. Each non-equilibrium dynamic has been propagated for a total of 20 ns to ensure complete equilibration. We have clustered the data obtained from all trajectories by extracting all the snapshots in a specific time interval. Merging data from different independent trajectories allows us to obtain a statistically significant average that is representative of the macroscopic system over a specific timeframe. From those grouped trajectories, we have computed the radial distribution function to check the evolution of the most relevant solute to the solvent interactions. This mainly involves the interactions of the oxygen O_2_, Oh, and Oa from the chromophore side, which account for the largest changes in S_0_/S_1_ point charges (see Section S5 in the Supplementary Materials). As concerns the solvent, unsurprisingly, the protic protons of water and ethanol stand out as the most relevant contributors. Interestingly, some structured interactions with the CN group of acetonitrile have been observed and may play a role.

**Figure 6:**
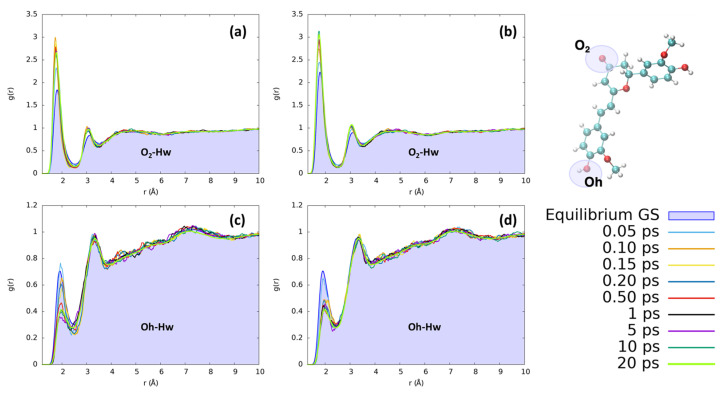
Radial distribution functions g(r), between the selected dye’s atoms (see the top right inset) and water hydrogens (Hw), computed at different time intervals before and after excitation along the MD trajectories. (**a**) CYC, O_2_-Hw; (**b**) PYR, O_2_-Hw; (**c**) CYC, Oh-Hw; and (**d**) PYR, Oh-Hw.

In the case of water, the most notable changes involve the hydroxyl group of the isomerizable phenyl ring and the O_2_ moiety. The observed changes are not extreme, but they clearly point to a restructuration of the solvation sphere, particularly affecting the first solvation shell, i.e., the regions where the solvent is more structured. The solvent reordering is extremely fast, occurring in less than 1 ps, as can be seen in Figure 6. Furthermore, the solvent reorganizes faster in the case of CYC, where the convergence of the peaks is reached in only 0.2 ps. Indeed, the short distance peak between the keto oxygen (O_2_, see Figure 1) and the water hydrogens increases very rapidly for CYC between 0.1 and 0.2 ps, before stabilizing around the equilibrium value. Such strong perturbation, induced by the electronic transition mimicked by the FF switch, may be associated with a very fast response of the CYC’s solvation sphere.

Conversely, in the case of PYR, the water molecules move slightly slower, resulting in a more progressive reorganization. The behavior of water around the OH group on the isomerizable ring is also remarkable and undergoes the opposite behavior than the one observed for O_2_. Indeed, in this case, the reorganization around PYR is much faster, while the solvent is rearranged at a slower rate around CYC, as shown in Supplementary Materials.

However, in both cases, the excitation leads to an increase in the number of solvent molecules involved in direct coordination with the keto group C_2_=O_2_ and induces a contraction of the first and second solvation shells, which are both clearly structured. Oppositely, the time evolution of the solvent around Oh points to a signifciant loss of structure within the first solvation shell, as shown by the significant decrease in the corresponding peak in the radial distribution function.

**Figure 7:**
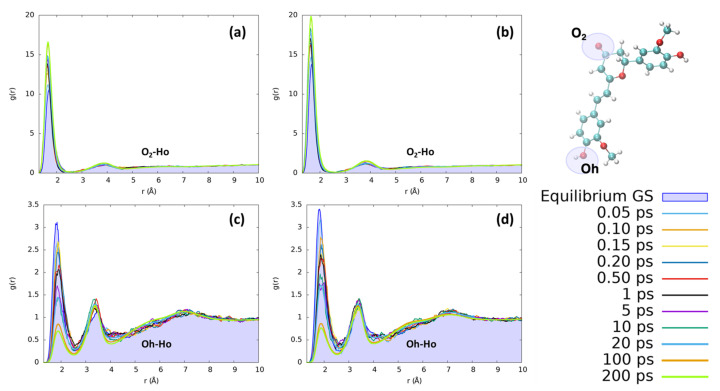
Radial distribution functions g(r) between the selected dye’s atoms (see the top right inset) and ethanol hydrogens (Ho), computed at different time intervals before and after excitation along the MD trajectories. (**a**) CYC, O_2_-Ho; (**b**) PYR, O_2_-Ho; (**c**) CYC, Oh-Ho; and (**d**) PYR, Oh-Ho.

The reorganization of ethanol around the same CYC and PYR atoms (O_2_ and Ho) does not present significant differences with respect to the features found for water. As can be seen in Figure 7, the solvation dynamics are very similar for both solvents, which undergo a contraction of the first neighbor shell upon excitation around O_2_ and the destructuration of the hydrogen bond network around Oh.

The first solvation shell peaks practically at the same distances as for water, while the second one is more distant (3.8 vs. 3.0 Å) due to both the larger size of ethanol with respect to water and the single proton of ethanol. Comparing the solvent reorganization around Oh, it appears that this group undergoes a stronger solvation in the ground state with respect to water, as can be seen from the more peaked radial distribution function. During the excited state solvent reorganization, however, the solvent is depleted from the first solvation shield, as can be seen by the decrease in the corresponding peak’s intensity. This effect may be due to the observed decrease in the RESP charge of oxygen (−0.71 to −0.63 for CYC and −0.74 to −0.66 for PYR), which makes it less electronegative and, thus, decreases the strength of hydrogen-bond interactions. Nonetheless, the major difference between the two solvents is due to the significantly slower dynamics registered in the ethanol response to excitation. Indeed, while in the case of water the full equilibration was achieved in 1 ps, ethanol requires a much longer time and reaches convergence after more than 100 ps. Besides being connected to the larger mass of ethanol with respect to water, the slower response of the former solvent can also be traced back to the weaker hydrogen-bond interactions settled by both dyes with the alcohol, and it is in line with previous findings concerning the response to the electronic excitation of similar chromophores embed in alcoholic environments [60,65,66,67].

**Figure 8:**
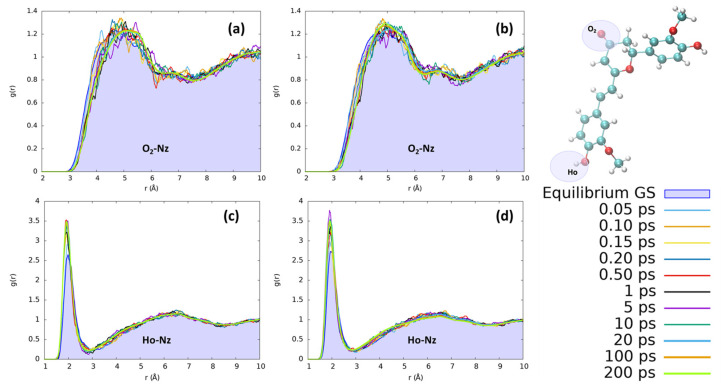
Radial distribution functions g(r), between the selected dye’s atoms (see the top right inset) and acetonitrile nitrogen (Nz), computed at different time intervals before and after excitation along the MD trajectories. (**a**) CYC, O_2_-Nz; (**b**) PYR, O_2_-Nz; (**c**) CYC, Ho-Nz; (**d**) and PYR, Ho-Nz.

In acetonitrile, the reorganization effects for both chromophores are less evident than those observed for protic solvents. In Figure 8, we have monitored the interaction with the CN nitrogen, which represents the moiety most suitable to interact with the solute due to its stronger point charges. Yet, observing the distribution of acetonitrile nitrosyl N around the hydrogen of the isomerizable phenyl ring, we clearly observe a less structured solvation scheme with respect to the ones obtained for the protic solvents. Interestingly, we found an increase in the number of acetonitrile interactions with Ho during the excited state solvent relaxation, as can be seen by the steadily increase in the first solvation shell peak at 2 Å. However, no evident structuring, for both CYC and PYR, can be observed beyond the first shell, since only a small and very broad peak around 6.5 Å can be observed. Both chromophores, i.e., CYC or PYR, behave exactly in the same way with respect to the reorganization of acetonitrile. Moving to the solvation around O_2_ is expected to have a long-range solvation and poor structuration, due to the impossibility of O⋯N interactions, because of the negative point charge on both atoms. While this is confirmed, we may see that in the excited state, the absolute value of the charge on O_2_ is increased, producing an effect that is translated into the elongation of the solvation sphere.

## 3. Methodology

Following the standard Joyce procedure [57,58], the generation of the QMD-FFs stands on the generation of selected QM descriptors. To this end, we have optimized both the chromophore’s the structure at the corresponding ground and the excited state minima, and we computed the corresponding Hessian matrices in a local harmonic approximation. The most flexible and anharmonic degrees of freedom were instead sampled using relaxed scans, to properly describe the potential energy surface (PES) of each system. All the QM calculations were carried out at DFT level for S_0_, while resorting to its time-dependent extension (TD-DFT), when tackling the excited state. In all cases, the CAM-B3LYP [68]/6-311++G** level of theory was used, to reach a consistent description of both states in the two considered chromophores. For all these calculations, three roots were computed at the TD-DFT level. In contrast, for the isomerizable coordinate, we have used the previously computed profiles at MS-CASPT2//CASSCF/6-31G*. For that, a state average between the 4 states used was included. The used active space was benchmarked and proved by previous studies, and it consists of 10 electrons in 9 orbitals [31,34].

The S_0_ and S_1_ QMD-FF parameterizations were carried out by exploiting the classical partition on the total energy of the system in an intra-molecular term, which rules the chromophore flexibility, and an intermolecular one, which governs its interaction with the solvent. The former term, consisting in the standard sum of stretching, bending, dihedral torsions and intra-molecular non-bonded contributions, was parameterized using the Joyce software G16, C01 [57,58], by minimizing the least squared differences between the corresponding QM and FF descriptors, namely the Hessian matrix elements and the relaxed torsional energy scans (see Supplementary Materials for further details). The inter-molecular term is instead expressed by a sum of Lennard-Jones (LJ) contributions plus Coulomb charge–charge interactions, where the LJ parameters have been directly transferred from the OPLS database, whereas the point charges were specifically modeled on the QM electronic density of each state/chromophore; these were computed while accounting for the different solvents at the PCM level, by applying the Restrained Electrostatic Potential (RESP) scheme, using Gaussian16 C.01 software [69] and the Antechamber 17.3 package [70]. Finally, as far as the solvent is concerned, the TIP3P model was adopted for water [71], while the generalized amber force field (GAFF) [72] was employed for ethanol and acetonitrile. In building all the QMD-FFs, the same model potential functions used in standard FFs were employed; hence, the computational cost of the successive MD runs is the same that could be expected from a simulation based on traditional FFs. It might also be worth noticing that while the parameterization of the intra-molecular term is performed once and for all on the isolated chromophore; the point charges entering the inter-molecular term are parameterized over the QM electron density, which accounts for the specific solvent at the PCM level, and hence should be recomputed for each considered new solvent. Further details can be found in the Supplementary Materials.

To build the initial system, each chromophore in its ground state was soaked in a periodic box with the investigated solvents, employing 1182, 972, and 987 molecules, for water, ethanol, and acetonitrile, respectively. Subsequently, the solvent has been relaxed following a three-step strategy, using the Gromacs 2019 package [73,74]. Firstly, the solute was constrained, and the solvent equilibrated for 1 ps to avoid close contacts. In a second step, the system is further equilibrated under NVT conditions for 1 ns. Finally, after a further equilibration step of 5 ns under NPT conditions, a production run at 300 K and 1 atm was performed for a total of 50 ns. Importantly, we have systematically saved coordinates and velocities to be used as a starting point for the subsequent non-equilibrium dynamics, concretely employing a series of 500 uncorrelated snapshots as initial conditions to sample the solvent reorganization upon excitation to the chromophore’s singlet excited state. To perform neqMD simulations, and to compute vertical transitions following the Born–Oppenheimer approximation, we maintained the ground state velocities and coordinates, while switching the QMD-FF of the chromophore to the one describing the targeted excited state. This procedure mimics the effect of light absorption, which induces a fast electronic reorganization followed by a slower geometrical movement. Afterwards, neqMD runs were extended up to 20 ns, i.e., a long enough time to ensure a full equilibration of the solvent to the new electronic distribution of the chromophore. For both the ground and excited state dynamics, a time step of 0.1 fs has been chosen to solve the Newton equations of motion, as no constraints were imposed on the bond distances. All the dynamics have been performed using the Gromacs package, using a v-rescale thermostat coupled with a Parrinello–Rahman method [73,74]. For the electrostatic and van der Waals interactions, a cut-off of 1.2 was used. All the production trajectories were run under the NPT ensemble. For the analysis of the solvent reorganization, we have computed the radial distribution function of crucial molecular moieties at different times, averaging along the independent 500 trajectories to assure statistical significance. The computed data were smoothed to facilitate the readability and reduce the noise using a 3-point running average strategy. Finally, the absorption spectra in different solvents were obtained by employing a sequential MD/QM scheme, based on a classical ensemble average of vertical energies (CEA-VE) [61], consisting in calculating, at the TD-DFT level, the vertical excitation energies for each of the 500 snapshots, eventually recovering the final spectral shape by averaging each stick transition, broadened with a gaussian function of HWHM = 0.15 eV. For this aim, CAM-B3LYP/6-311++G** was used for consistency with the QMD-FF, and a total of 10 states have been calculated for each snapshot.

## 4. Conclusions

In the present contribution, we have used QMD-FFs obtained following the Joyce procedure, to model the optical properties of two biomimetic photoswitches which can possibly be used for LAC purposes, solvated by different solvents of increasing polarity and proticity. Indeed, the interplay between electronic excitation and solvent relaxation is fundamental to dictate the following photophysical and photochemical response and should be correctly modeled. For this aim, the use of QMD-FFs is particularly attractive, since it allows us to model the potential of a specific excited state, and hence run non-equilibrium simulations to describe the evolution in the excited state manifold.

After the precise validation of the QMD-FF for both chromophores, we have shown that our procedure is able to retrieve all the experimental properties; in particular, the slight but peculiar solvatochromism observed for acetonitrile is perfectly recovered. Finally, we have shown significant differences in the solvent relaxation, concerning the equilibration characteristic time between water and ethanol, as well as the redistribution of the first-shell solvation molecules. Conversely, while acetonitrile shows less structured interactions, some non-negligible changes in the distribution of the nitrosyl CN group around the polar moieties of the chromophore can be envisaged.

Our work, while showing the potentiality of QMD-FF procedures in modeling subtle photophysical and photochemical properties, provides a first atomistically resolved description of the solvent reorganization around organic chromophores. In particular, the global effects of the polarity, as well as those due to directional and specific effects, such as hydrogen bonds, are retrieved. Furthermore, the validation of the QMD-FF provided in this contribution may also allow us to proceed to the study of the photoisomerization process by using purely classical methods, and thus achieve much longer time scales. Although our work remains fundamental, its outcome, and in particular the elucidation of the coupling between photoexcitation and solvent relaxation, may also have important consequences for the optimization of curcumin-based systems for phototherapeutic purposes. Indeed, such systems would target strongly inhomogeneous environments, such as lipid bilayers and membranes, which present a coexistence of hydrophobic and polar regions. Knowing how the solvent reorganizes in the early phases of the response to light stimuli is therefore important to precisely tune the further relaxation channels, for instance photoisomerization, which are susceptible to drive a biological and therapeutic response.

## Data Availability

The data presented in this study are available in article and Supplementary Materials.

## Supplementary Materials

The following supporting information can be downloaded at: https://www.mdpi.com/article/10.3390/molecules29081752/s1, Additional details on the QMD-FF parameterization, potential energies surfaces for the coupled dihedral, comparison between the QMD-FF and QM scans, and vibrational frequencies. NTOs for both compounds. Point charges for the different solvents.
