# Supplementary material for: A Portrait of the Chromophore as a Young System—Quantum-Derived Force Field Unraveling Solvent Reorganization upon Optical Excitation of Cyclocurcumin Derivatives
Source: Molecules. 2024 Apr 12;29(8):1752. doi: 10.3390/molecules29081752 (PMC11052401; doi:10.3390/molecules29081752)
Supplement: Supplementary file 1 [file molecules-29-01752-s001.zip › molecules-2879232-supplementary.pdf]

S1] QMD-FF parameterization

The QMD-FF for a solvated system composed by either the CYC or PYR chromophore and a number (N_{solv}) of solvent molecules (either water, ethanol oe acetonitrile) is partitioned in one inter- and one intra-molecular term:

$$E_{tot}^{QMD-FF} = E_{intra}^{QMD-FF} + E_{inter}^{QMD-FF} \quad \text{(S1)}$$

E_{inter}^{QMD-FF}drives the solute's interaction with the solvent and the interactions among solvent molecules, whereas E_{inter}^{QMD-FF}rules solute and solvent 's flexibility, i.e.

$$E_{intra}^{QMD-FF} = E_{CYC/PYR}^{QMD-FF} + E_{solv}^{QMD-FF} \quad \text{(S2)}$$

The solute-solvent and solvent-solvent interactions are instead accounted for by the first term of equation (S1), that is

$$E_{inter}^{QMD-FF} = \sum_{i=1}^{N_{solu}} \sum_{j=1}^{M_{solv}} E_{ij}^{solu-solv} + \sum_{i=1}^{M_{solv}} \sum_{j=1}^{M_{solv}} E_{ij}^{solv-solv} \quad \text{(S3)}$$

where N_{solu} and N_{solv} are the number of solute (CYC or PYR) and solvent (water, ethanol or acetonitrile) atoms, respectively, and $E_{ij}^{solu-solv}$and $E_{ij}^{solv-solv}$are computed as the standard sum of a 12-6 LJ potential and Coulomb charge-charge interactions:

$$E_{ij}^{x-solv} = 4\epsilon_{ij}\left[\left(\frac{\sigma_{ij}}{r_{ij}}\right)^{12} - \left(\frac{\sigma_{ij}}{r_{ij}}\right)^{6}\right] + \frac{q_i q_j}{r_{ij}} \quad \text{(S4)}$$

where *x* stands for either *solu* or *solv*. As far as the solvent-solvent interactions and the solvent flexibility are concerned, as detailed in the main text, the TIP3P model was adopted for water, while the parameters describing ethanol and acetonitrile were transferred from the generalized amber force field (GAFF).

Conversely, the FF terms concerning with the solute-solvent interaction and the chrompohore's intramolecular term were refined specifically for the target system: the former intermolecular term, $E_{ij}^{solu-solv}$, was refined by deriving through the RESP scheme the solute point charges entering in eq. (S4) from QM calculations, purposely carried out on the solute's optimized geometries in either their ground or excited state. Concretely, all CYC or PYR atomic charges were derived from CAM-B3LYP/6-311++G** electronic density computed at DFT (for the ground state) or TD-DFT (for S_1), accounting for the solvent by means of the C-PCM method. Finally, the LJ terms entering eq. (S4) were transferred for both states from the OPLS FF.

The intramolecular term $E_{CYC/PYR}^{QMD-FF}$was derived through the JOYCE protocol from the QM database consisting in the gas phase optimized geometry, its Hessian matrix and the relaxed torsional energy scans. Notwithstanding all the details of JOYCE procedure can be

found in the original papers, a brief description of the specific parameterization of the ground and excited states of both CYC and PYR chromophores is given in the following. Both the intramolecular QMD-FF terms for the ground and excited state (S_1) of the solute (CYC or PYR) take the standard expression:

$$E_x^{QMD-FF} = E_s + E_b + E_{ht} + E_{ft} + E_{inb} \tag{S5}$$

where the first three terms refer to stretching (E_s), angle bending (E_b) and distortions of harmonic dihedrals (E_{ht}), depend on stiff redundant internal coordinates, and are therefore approximated through harmonic potentials:

$$E_s = \frac{1}{2}\sum_i^{N_{bonds}} k_i^s (r - r_0)^2 \tag{S6}$$

$$E_b = \frac{1}{2}\sum_i^{N_{angles}} k_i^b (\theta - \theta_0)^2 \tag{S7}$$

$$E_{ht} = \frac{1}{2}\sum_i^{N_{dihedrals}^{stiff}} k_i^{ht} (\varphi - \varphi_0)^2 \tag{S8}$$

Conversely, E_{ft} depends on the soft dihedrals δ, which are expected to present an enhanced flexibility and are hence represented by Fourier-like expansions:

$$E_{ft} = \sum_{\mu}^{N_{dihedrals}^{flex}} \sum_j^{N_{cos_\mu}} k_{j\mu}^{ft}\left(1 + cos\left[n_{j\mu}\delta_\mu - \gamma_{j\mu}\right]\right) \tag{S9}$$

Finally, the last term of equation (S5) also contributes to the internal energy of the chromophore, and again depends on soft internal coordinates, namely the intra-molecular distances between selected CYC (or PYR) atoms:

$$E_{inb} = \sum_{i<j}^{N_{inb}} 4\epsilon_{ij}^{intra}\left[\left(\frac{\sigma_{ij}^{intra}}{r_{ij}}\right)^{12} - \left(\frac{\sigma_{ij}^{intra}}{r_{ij}}\right)^{6}\right] \tag{S10}$$

It is worth mentioning that according to the JOYCE protocol intra-molecular nonbonded LJ parameters ε_{ij}^{intra} and σ_{ij}^{intra} are allowed to take different values from the ones employed for the description of inter-molecular interactions (ε_{ij} and σ_{ij}) entering in equation (S4), and they allow for a more specific parameterization since they can be included in the QMD-FF just for selected chromophore's atom pairs.

All other intramolecular QMD-FF parameters were obtained with the JOYCE code, by performing the usual two-step procedure: a first cycle which fits all harmonic parameters at once and a second cycle, in which the harmonic parameters are fixed and the parameters for the remaining flexible dihedrals are parameterized against the QM scans. The same procedure was followed for the ground and the excited state.

S2] QMD-FF validation

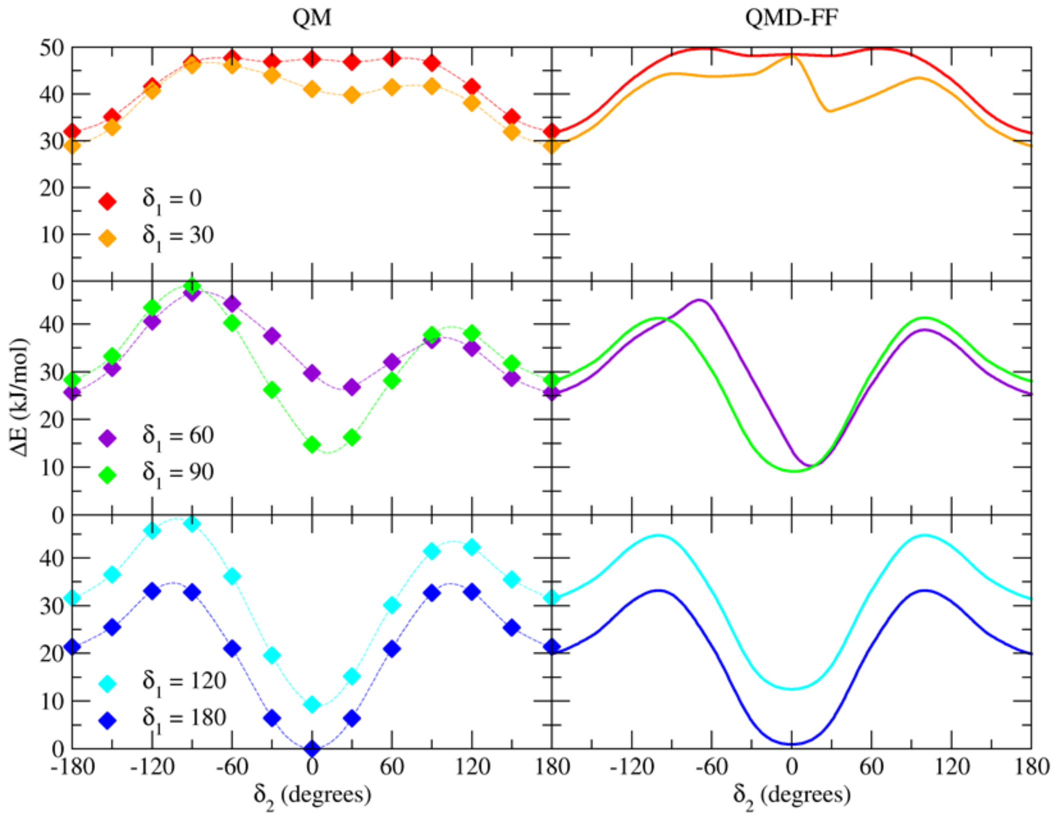

Figure S1. Coupled dihedrals δ_1 and δ_2.

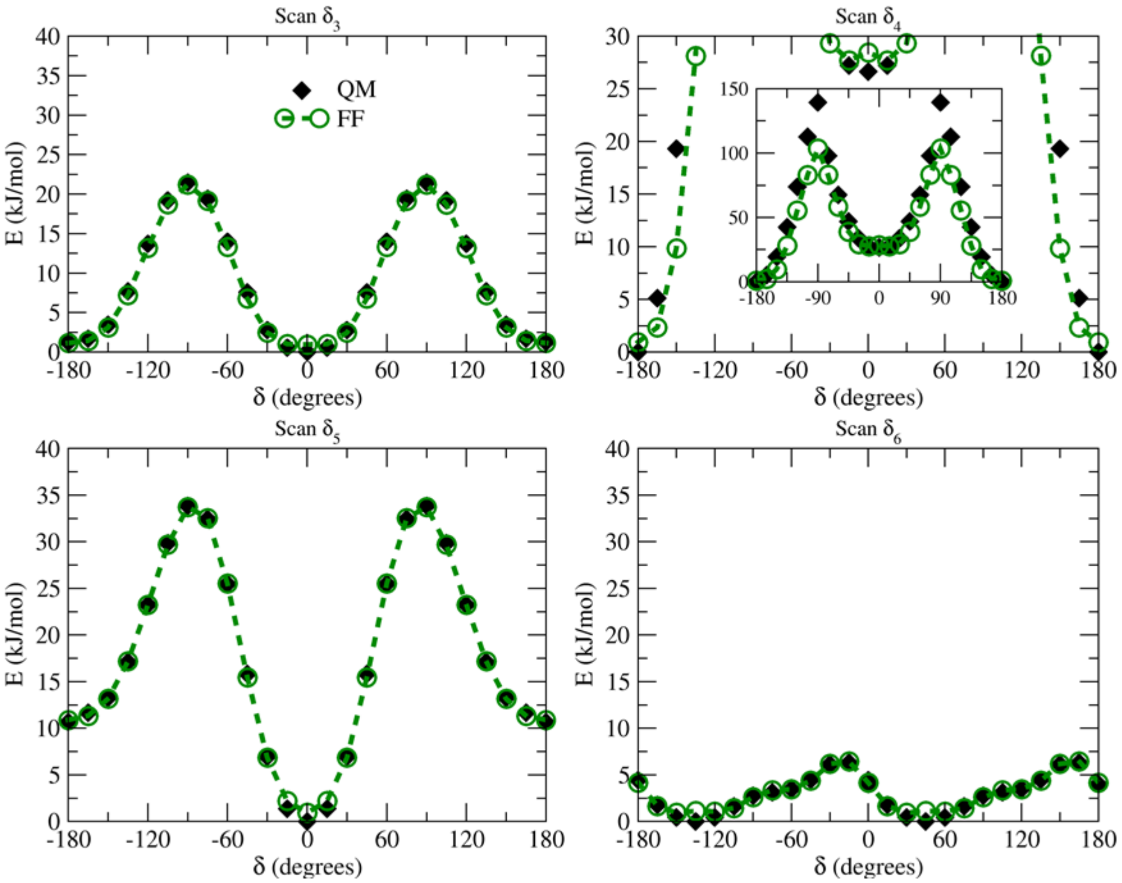

Figure S2. Comparison between QM and QMD-FF scans for the relevant IC of Cyclocurcumin (CYC) on ground state.

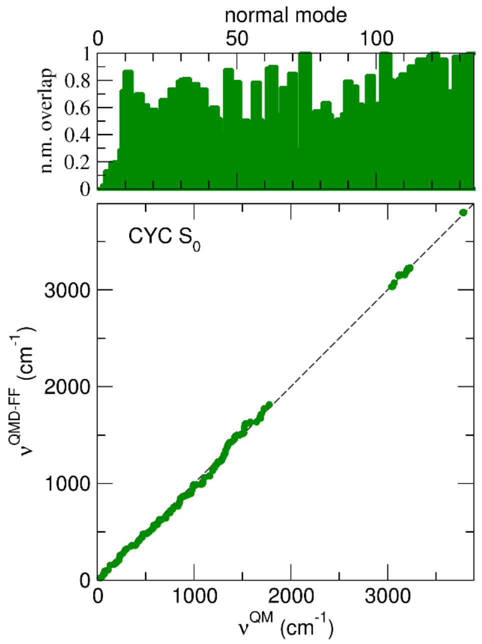

Figure S3. Comparison between QM and QMD-FF normal modes for Cyclocurcumin (CYC) on ground state.

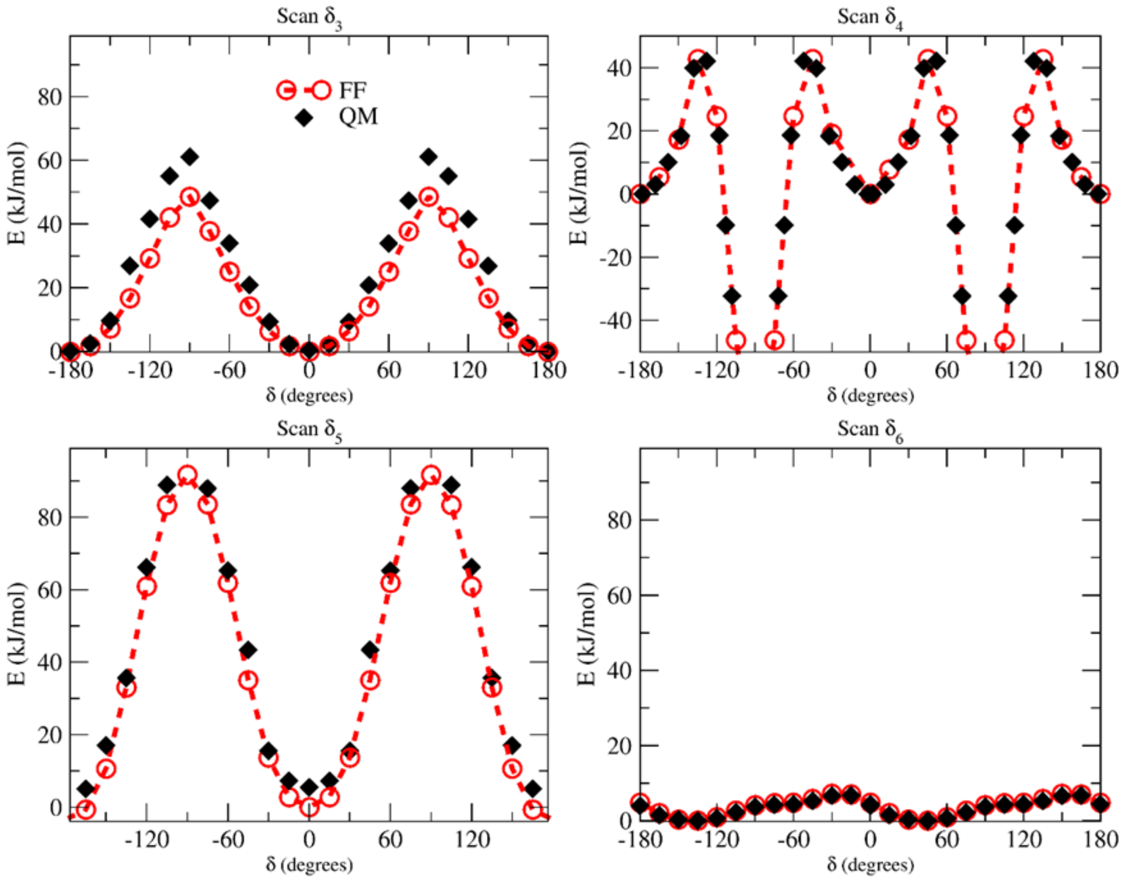

Figure S4. Comparison between QM and QMD-FF scans for the relevant IC of Cyclocurcumin (CYC) on π-π excited state.*

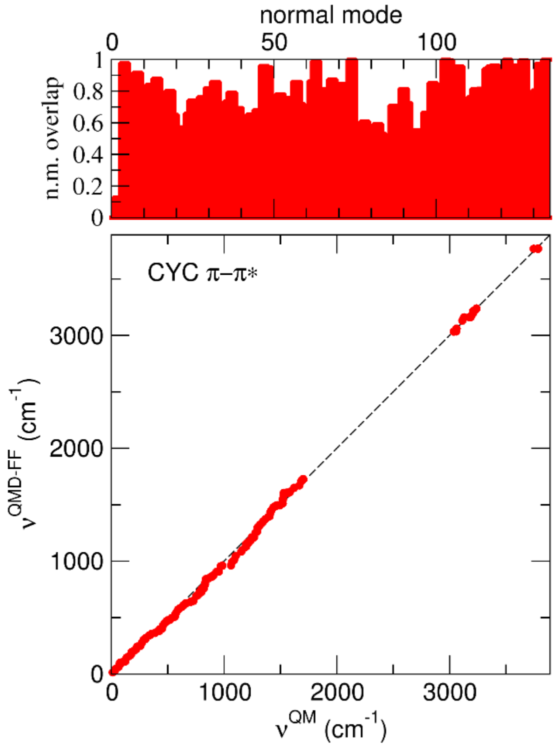

Figure S5. Comparison between QM and QMD-FF normal modes of Cyclocurcumin (CYC) on π-π excited state.*

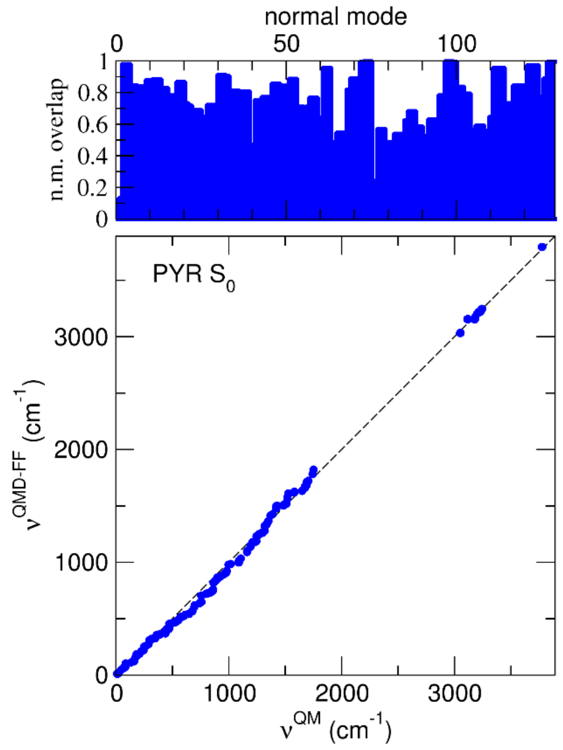

Figure S6. Comparison between QM and QMD-FF normal modes of Pyron (PYR) on Ground state.

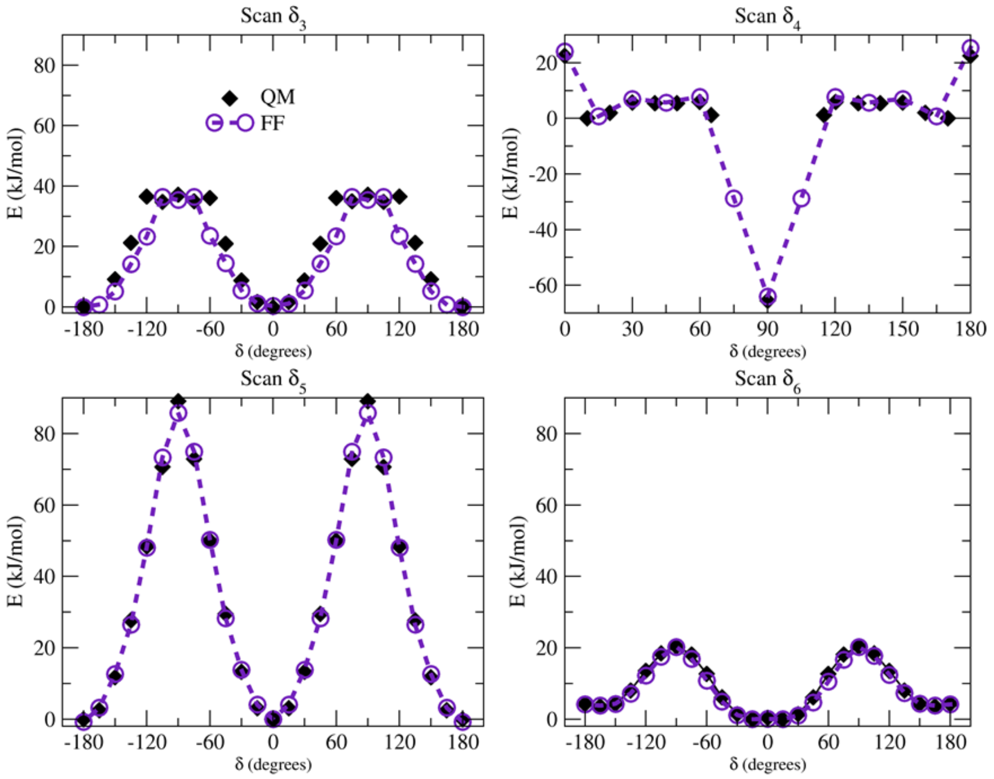

Figure S7. Comparison between QM and QMD-FF scans for the relevant IC of Pyron (PYR) on π-π excited state.*

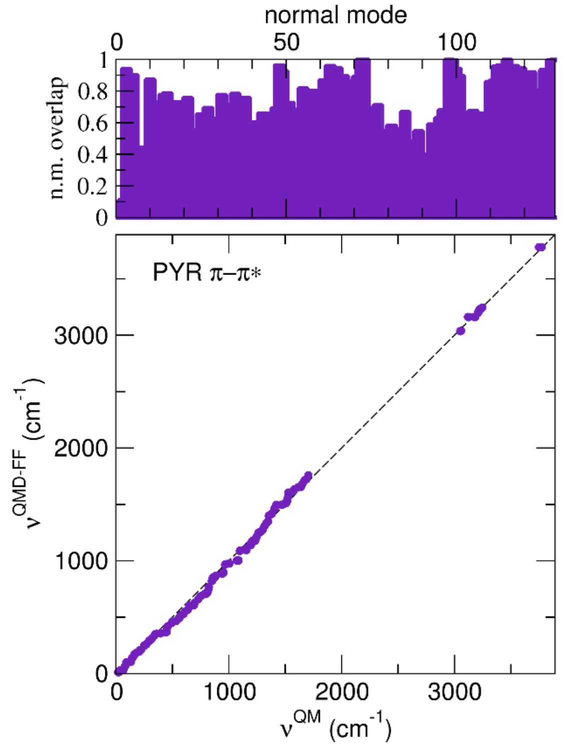

Figure S8. Comparison between QM and QMD-FF normal modes of Pyron (PYR) on π-π excited state.*

S3] MD conformational analysis

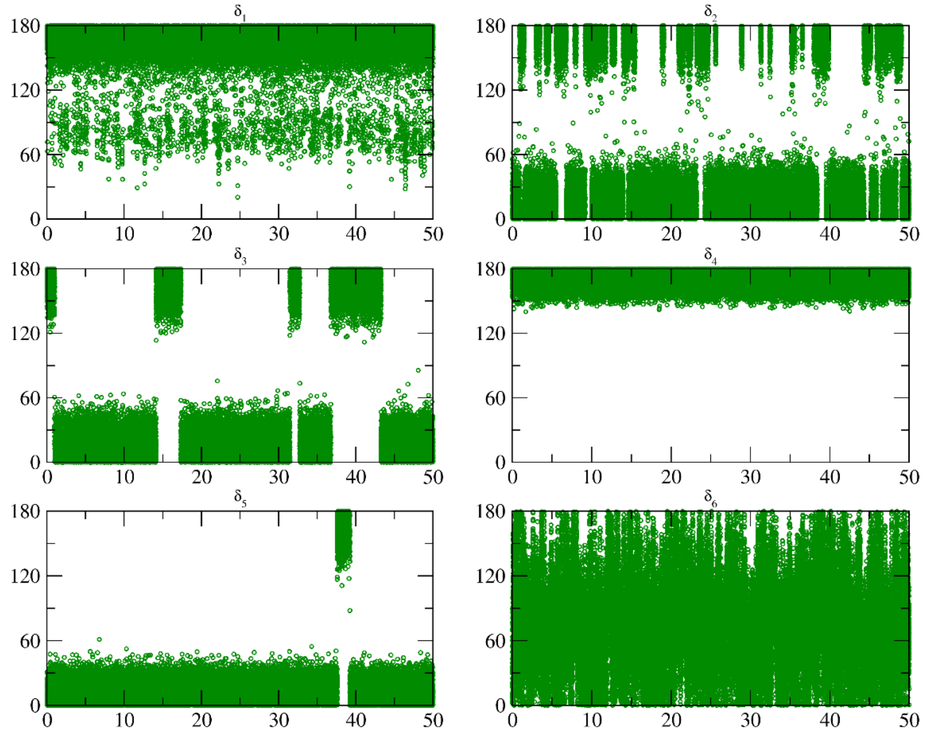

Figure S9. Temporal evolution of all the scanned dihedrals for CYC in water. Absolute value of the dihedral vs. time in ns.

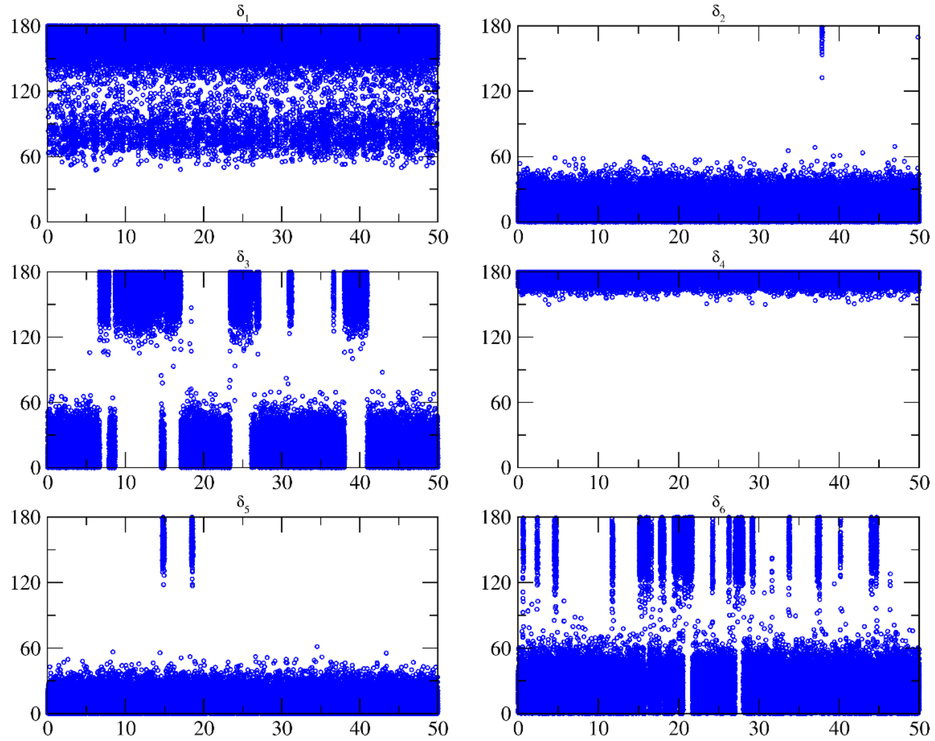

Figure S10. Temporal evolution of all the scanned dihedrals for PYR in water. Absolute value of the dihedral vs. time in ns.

S4] Natural Transition Orbitals

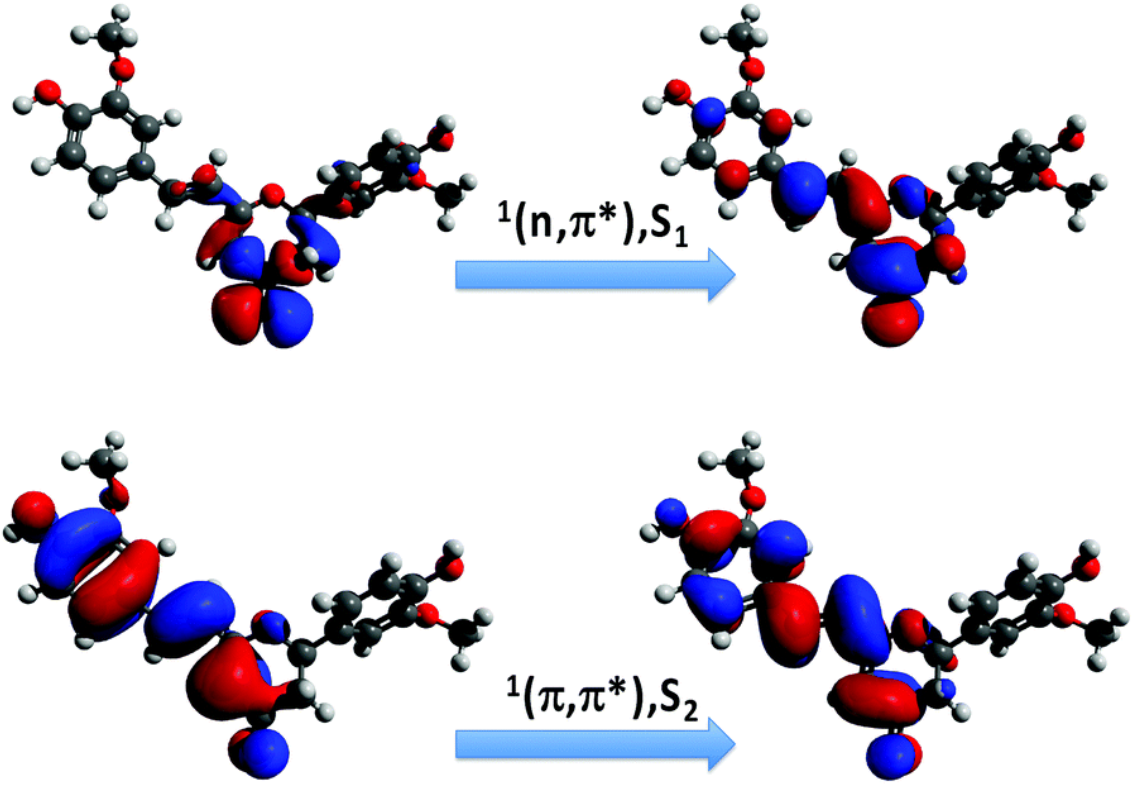

Figure S11. NTOs of CYC for the first two excited states found in gas phase at CAM-B3LYP/6-31G* level. Reproduced from Phys.Chem.Chem.Phys., 2020, 22, 4749.

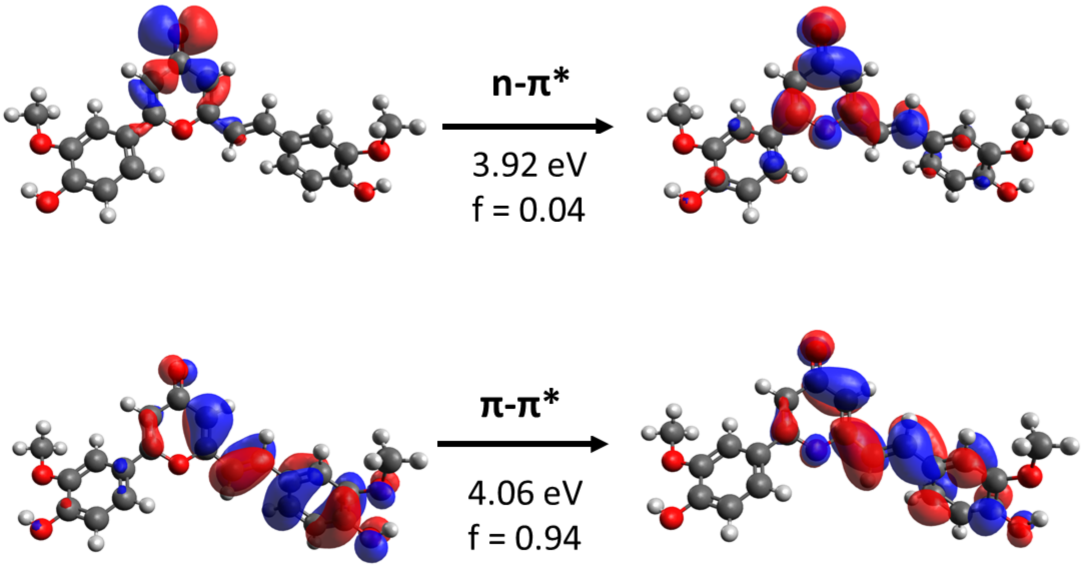

Figure S12. NTOs of PYR for the first two excited states found in gas phase at CAM-B3LYP/6-31G* level. Reproduced from Phys. Chem. Chem. Phys., 2021,23, 12842-12849.

S5] Point charges for the different solvents

	Water		Ethanol		Acetonitrile	
	CYC GS	CYC π-π*	CYC GS	CYC π-π*	CYC GS	CYC π-π*
OS	-0.163932	-0.121631	-0.162716	-0.120796	-0.163225	-0.121169
Cv0	0.108310	0.038707	0.107130	0.038789	0.107597	0.038758
Cv1	-0.348525	-0.385770	-0.347542	-0.383153	-0.347940	-0.384309
C_2	0.524171	0.499607	0.522760	0.498150	0.523364	0.498794
Ch2	-0.030782	-0.012160	-0.030793	-0.012043	-0.030777	-0.012087
Ch	0.091813	0.091699	0.090654	0.090651	0.091161	0.091116
Hv1	0.165321	0.179245	0.164995	0.178760	0.165132	0.178976
Hc2	0.068554	0.055377	0.068441	0.055519	0.068492	0.055456
Cv2	-0.231992	-0.186388	-0.231681	-0.186426	-0.231785	-0.186410
Cv3	-0.024586	-0.091629	-0.024707	-0.091549	-0.024662	-0.091581
Hv2	0.187497	0.180613	0.186779	0.179964	0.187092	0.180249
Hv3	0.112374	0.088258	0.112338	0.087938	0.112352	0.088077
Ca1	-0.069454	0.001511	-0.069166	0.001637	-0.069325	0.001580
Ca2	-0.154291	-0.176937	-0.153901	-0.177036	-0.154044	-0.176994
Ca3	-0.213870	-0.172073	-0.213551	-0.172954	-0.213644	-0.172573
Ca4	-0.243918	-0.226919	-0.242983	-0.226399	-0.243456	-0.226624
Ca5	0.258778	0.319026	0.258366	0.317493	0.258602	0.318161
Ca6	0.150278	0.160594	0.149868	0.160274	0.150011	0.160422
Ha2	0.145546	0.174044	0.144749	0.173318	0.145095	0.173639
Ha3	0.190534	0.172766	0.190070	0.172383	0.190268	0.172556
Ha4	0.192986	0.196604	0.192707	0.196268	0.192844	0.196417
Ca7	-0.108858	-0.169161	-0.108524	-0.168454	-0.108722	-0.168770
Ca2	-0.129365	-0.106531	-0.129126	-0.106561	-0.129190	-0.106550
Ca8	-0.296475	-0.233281	-0.296169	-0.233203	-0.296274	-0.233237
Ca4	-0.147286	-0.181372	-0.146255	-0.180381	-0.146734	-0.180818
Ca5	0.200032	0.219128	0.199916	0.218928	0.199999	0.219014
Ca6	0.148035	0.122205	0.147579	0.121978	0.147745	0.122077
Ha2	0.146457	0.146129	0.145624	0.145495	0.145987	0.145777
Ha8	0.192173	0.168133	0.191797	0.167925	0.191958	0.168018
Ha4	0.173576	0.177901	0.173332	0.177552	0.173443	0.177705
Oa	-0.241490	-0.233259	-0.240953	-0.232891	-0.241187	-0.233052
CT	-0.125642	-0.150543	-0.125380	-0.150260	-0.125485	-0.150377
HT	0.104436	0.110935	0.104069	0.110556	0.104228	0.110722
HT	0.104436	0.110935	0.104069	0.110556	0.104228	0.110722
HT	0.104436	0.110935	0.104069	0.110556	0.104228	0.110722
Oh	-0.599827	-0.604975	-0.598334	-0.603614	-0.599003	-0.604216
Ho	0.426148	0.427122	0.425291	0.426267	0.425673	0.426645
Oa	-0.232427	-0.238346	-0.231915	-0.238148	-0.232137	-0.238238
CT	-0.156120	-0.124346	-0.156047	-0.124313	-0.156075	-0.124334
HT	0.117017	0.112923	0.116714	0.112660	0.116847	0.112779
HT	0.117017	0.112923	0.116714	0.112660	0.116847	0.112779

HT	0.117017	0.112923	0.116714	0.112660	0.116847	0.112779
O_2	-0.635840	-0.714937	-0.632705	-0.710059	-0.634090	-0.712215
Oh	-0.587567	-0.551190	-0.586115	-0.550670	-0.586771	-0.550902
Ho	0.419899	0.438344	0.419032	0.437414	0.419418	0.437827
Hc2	0.068554	0.055377	0.068441	0.055519	0.068492	0.055456
Hc	0.106852	0.097484	0.106347	0.097037	0.106573	0.097234

	Water		Ethanol		Acetonitrile	
	PYR GS	PYR π-π*	PYR GS	PYR π-π*	PYR GS	PYR π-π*
OS	0.027840	0.010015	0.027340	0.009811	0.027560	0.009898
Cv0	0.067068	0.073233	0.066353	0.072840	0.066670	0.073012
Cv1	-0.234942	-0.288375	-0.233776	-0.285132	-0.234289	-0.286561
C_2	0.516102	0.493048	0.514531	0.491335	0.515225	0.492094
Cv4	-0.225056	-0.215480	-0.224375	-0.214050	-0.224675	-0.214676
Cv5	0.105625	0.074376	0.105354	0.073925	0.105472	0.074124
Hv1	0.156001	0.164355	0.155656	0.163889	0.155808	0.164097
Hv4	0.150376	0.143149	0.150091	0.142991	0.150219	0.143061
Cv2	-0.252698	-0.269513	-0.252342	-0.269028	-0.252496	-0.269238
Cv3	-0.030667	-0.087795	-0.030429	-0.087673	-0.030537	-0.087728
Hv2	0.196276	0.202512	0.195755	0.201698	0.195985	0.202057
Hv3	0.081330	0.072712	0.080610	0.072026	0.080927	0.072326
Ca1	-0.025394	0.024857	-0.025329	0.024873	-0.025358	0.024864
Ca2	-0.166215	-0.200829	-0.166032	-0.200903	-0.166117	-0.200870
Ca3	-0.239283	-0.143567	-0.238833	-0.144374	-0.239034	-0.144022
Ca4	-0.224199	-0.271353	-0.223159	-0.270734	-0.223619	-0.271007
Ca5	0.269715	0.353286	0.269247	0.351695	0.269455	0.352395
Ca6	0.122205	0.144018	0.121976	0.143730	0.122078	0.143857
Ha2	0.171337	0.185037	0.170459	0.184199	0.170849	0.184570
Ha3	0.183403	0.163333	0.182857	0.162985	0.183100	0.163141
Ha4	0.190917	0.205135	0.190623	0.204802	0.190755	0.204949
Ca7	-0.086907	-0.044524	-0.086738	-0.044072	-0.086817	-0.044274
Ca2	-0.167999	-0.190932	-0.167940	-0.190429	-0.167967	-0.190650
Ca8	-0.229415	-0.251159	-0.228851	-0.250629	-0.229097	-0.250862
Ca4	-0.187474	-0.190563	-0.187240	-0.190511	-0.187348	-0.190544
Ca5	0.183965	0.230700	0.183771	0.230801	0.183858	0.230770
Ca6	0.209049	0.170764	0.208837	0.170198	0.208936	0.170444
Ha2	0.152989	0.167442	0.152184	0.166711	0.152542	0.167038
Ha8	0.187729	0.189250	0.187120	0.188579	0.187390	0.188879
Ha4	0.189477	0.187198	0.189174	0.186785	0.189310	0.186969
Oa	-0.242682	-0.240969	-0.242319	-0.240624	-0.242486	-0.240783
CT	-0.170059	-0.167974	-0.170200	-0.168101	-0.170138	-0.168041
HT	0.120435	0.120675	0.120261	0.120501	0.120338	0.120578
HT	0.120435	0.120675	0.120261	0.120501	0.120338	0.120578
HT	0.120435	0.120675	0.120261	0.120501	0.120338	0.120578

Oh	-0.575360	-0.588603	-0.574016	-0.587434	-0.574614	-0.587958
Ho	0.425624	0.429903	0.424752	0.429058	0.425141	0.429434
Oa	-0.238692	-0.234782	-0.238164	-0.234552	-0.238398	-0.234657
CT	-0.140042	-0.129604	-0.139934	-0.129635	-0.139979	-0.129626
HT	0.110165	0.113740	0.109850	0.113481	0.109989	0.113598
HT	0.110165	0.113740	0.109850	0.113481	0.109989	0.113598
HT	0.110165	0.113740	0.109850	0.113481	0.109989	0.113598
O_2	-0.668741	-0.748770	-0.664988	-0.743853	-0.666648	-0.746027
Oh	-0.600857	-0.559204	-0.599342	-0.558632	-0.600015	-0.558887
Ho	0.427856	0.436429	0.426985	0.435489	0.427372	0.435907
